# The Association between Patent Ductus Arteriosus and Perinatal Infection in A Group of Low Birth Weight Preterm Infants

**Published:** 2013-11-22

**Authors:** Edmond Pistulli, Arjan Hamiti, Sokol Buba, Alketa Hoxha, Nita Kelmendi, Gentian Vyshka

**Affiliations:** 1 Faculty of Technical and Medical Sciences, University of Tirana; 2 University Hospital Center “Mother Teresa”; 3 University of Prishtina, Kosovo; 4 Faculty of Medicine, University of Tirana, Albania

**Keywords:** Prematurity, Perinatal Infection, Patent Ductus Arteriosus, Oral Ibuprofen, Intravenous Ibuprofen

## Abstract

***Objective:*** Patent ductus arteriosus (PDA) is an extremely common occurrence in very premature infants. Untreated symptomatic PDA may be associated with chronic lung disease. PDA has a major role in neonatal mortality and morbidity. We compared the efficacy and safety of oral versus intravenous ibuprofen for the pharmacological closure of PDA in low birth weight (LBW) preterm infants.

***Methods:*** A randomized, single-blinded, controlled study was performed on premature neonates at the neonatal unit, University Hospital for Obstetrics and Gynecology “Koço Gliozheni”, Tirana, Albania from January 2010 to December 2012. The study enrolled 68 preterm infants with a confirmed and significant PDA. The preterm infants received either intravenous or oral ibuprofen randomly as an initial dose of 10 mg/kg, followed by 5 mg/kg at 24 and 48 h.

***Findings*** ***:*** 36 patients were treated with oral ibuprofen and 32 with intravenous ibuprofen during this period. After the first course of the treatment, the PDA closed in 30 (83.3%) of the patients assigned to the oral ibuprofen group versus 23 (71.8%) of those enrolled in the intravenous ibuprofen group (*P*=0.355). 15 patiens needed a second treatment course and they all (100%) had clinical signs of infection and positive blood culture. There was no reopening of the ductus after the closure.

***Conclusion:*** Our data indicate that, for LBW infants, the rate of early ductal closure was comparable and the adverse effects were fewer with oral ibuprofen in comparison to the intravenous route. Association of PDA with perinatal infection has a negative impact in pharmacological closure of the ductus, increasing the need for a second course of treatment or for surgery.

## Introduction

Patent ductus arteriosus (PDA) is extremely common in very premature infants and untreated symptomatic PDA may be associated with chronic lung disease^[^^1^^]^. Clinical and epidemiological data strongly suggest that infections, either prenatal or nosocomial, and the presence of a patent ductus arteriosus (PDA) play a major role in the neonatal mortality and morbidity^[^^2^^,^^3^^,^^4^^]^. For this reason, efforts to prevent this complication in low birth weight infants should include an aggressive approach to the prevention and treatment of prenatal and neonatal infections and an early closure of the PDA. Pharmacological closure of PDA with indomethacin or with ibuprofen, that are both prostaglandin inhibitors, has remained the mainstay of treatment in premature infants over the last three decades^[^^5^^,^^6^^]^. 

 During the search for an explanation of the interaction between neonatal infection and PDA, is observed that the presence of a systemic infection in the premature infant adversely affects permanent closure of the ductus, often inducing ductal opening after the first week of life and failure to respond to medical treatment with indomethacin^[^^7^^]^. A likely explanation for this interaction is the elevated serum levels of prostaglandins and tumor necrosis factor (TNF) observed in infants with infections. In addition, infants with serious infections frequently have complications that prevent or delay the medical or surgical treatment of the PDA. 

 As a result, the ductus remains open for prolonged periods of time, maintaining an increased pulmonary blood flow, high capillary pressure, and increased lung fluid. Furthermore, when both complications (infection and PDA) occurred at the same time, they produced a synergistic interaction, further increasing the risk for developing chronic lung disease (CLD)^[^^10^^]^. As a consequence of the left-to-right shunting through the PDA, pulmonary blood flow and lung fluid increases, negatively affecting lung function and gas exchange, and thereby increasing the risk for CLD^[^^9^^]^. The presence of a PDA has also been associated with elevated concentrations of myeloperoxidase in the tracheobronchial fluid, suggesting that the increased pulmonary blood flow may result in damage of the pulmonary endothelium and adhesion and migration of polymorphonuclear cells (PMNs) into the lung tissue^[^^7^^,^^8^^]^. Considerable biological plausibility thus exists to explain the influence of significant PDA and sepsis on feed tolerance in preterm neonates. PDA and sepsis are possibly markers of prematurity, and a prolonged interval between starting feeding and full enteral nutrition simply reflects the reluctance to start or continue feeding in the presence of such perceived risk factors for food intolerance and necrotizing enterocolitis (NEC)^[^^11^^,^^12^^]^.

 In this study, we compared the efficacy and safety of oral versus intravenous ibuprofen for the pharmacological closure of PDA in low birth weight (LBW) preterm infants.

## Subjects and Methods

The study was designed as a prospective, randomized, single-blinded study. The study was conducted in the neonatal intensive care unit of the University Hospital for Obstetrics and Gynecology ”Koço Gliozheni”, Tirana, Albania, between January 2010 to December 2012. This study was approved by the Medicine University and Neonatology Department.

 The study enrolled preterm infants with a gestational age 28-32 weeks, birth weight ≤2000g, postnatal age 48-96 hours, respiratory distress syndrome (RDS) treated with mechanical ventilation (CPAP [continuous positive airway pressure] or IPPV [intermittent positive pressure ventilation]) with additional oxygen requirements above 30% and one of the following echocardiographic criteria: a ductal size >1.5 mm, a left atrium-to-aorta ratio >1.4, and a left-to-right shunting of blood in addition to signs of PDA. Several sources have used a similar cutoff in the value of PDA, with inclusion of patients whose ductus’ diameter was more than 1.5 mm, although no general consensus is achieved^[^^4^^-^^6^^]^. 

 2D (two-dimensional) echocardiography was performed with an ALOKA ultrasound machine (Hitachi), with 5 and 7.5 MHz electronic sector transducers. Gestational age (GA) was assessed by obstetrical dating criteria or, when obstetrical data was inadequate, by Ballard examination. 

**Fig. 1:**
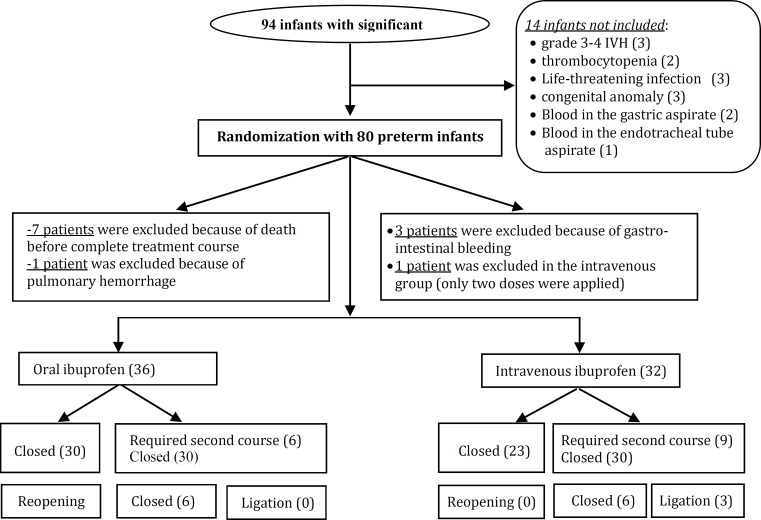
Flow chart of the study

 Exclusion criteria were major congenital abnormalities, right–to–left ductal shunting, life-threatening infection, grade 3 or 4 intraventricular hemorrhage, oliguria of less than 1 ml/kg/h during the preceding eight hours, serum creatinine concentration in excess of 1.6 mg/dl, blood urea nitrogen in excess of 60 mg/dl, thrombocyte count of less than 60 000/mm^3^, clinical bleeding tendency as revealed by hematuria, blood in the gastric aspirate or in the stools, blood in the endotracheal tube aspirate, oozing from venous or capillary puncture sites, hyperbilirubinemia for which exchange transfusion was required and pulmonary hypertension (Fig. 1).

 All infants who met the inclusion criteria first underwent echocardiography and cranial ultrasonography, after which they were treated with oral ibuprofen (Brufen, Abbot S.r.l; Italy). Ibuprofen in a dose of 10 mg/kg was given via an orogastric tube, flushed with 1 mL of sterile water to ensure delivery of the drug, otherwise an intravenous route was used (Pedea, Orphan Europe; a vial of 2 mL containing 10 mg of ibuprofen), with ibuprofen infused over a 15-minute period with a syringe pump, and the line was subsequently flushed with saline. 

 The two imaging procedures were performed again 24 hours after each ibuprofen dose. When the PDA was still hemodynamically significant, as demonstrated by echocardiography, and there was no evidence of deterioration in brain ultrasonography, a second dose of ibuprofen 5 mg/kg was administered. A third equivalent dose was given after another 24 hours if deemed necessary. Cranial ultrasound was repeated one week after the last ibuprofen dose and again before discharge from the ward. 

 RDS was treated with respiratory support (CPAP, IPPV or with high-frequency ventilation), oxygen supplements, and surfactant (Curosurf, Chiesi, Italy; a vial of 1.5 mL containing 120 mg) was administered intratracheally at the dosage of 100 to 200 mg/kg. Prophylactic antibiotics were started on admission and stopped after five days if blood cultures were negative. 

 Occurrence of any of the following conditions was enough to discontinue treatment: IVH intraventricular hemorrhage (IVH) grade 3–4, renal failure, NEC, and presence of GEB (gastrointestinal bleeding). 

 Before and 24 hours after treatment, all patients were evaluated with a complete blood count, renal function tests (serum creatinine level, blood urea nitrogen and urine output), cranial ultrasonography, and echocardiography. All infants continued their current enteral feeding during the treatment.

## Findings

A total of 168 premature infants at gestational age <32 weeks and birth weight <2000g and RDS were admitted to our NICU (neonatal intensive care unit), from January 2010 to December 2012 and underwent an echocardiographic Doppler ultrasound evaluation at the age of 48-96 hours. The entire study protocol was completed for 80 patients due to drop-out related to various reasons (Fig. 1).

 We had a minimum value of PDA of 1.5 mm (as inclusion criteria) and a maximum of 3.4 mm (average 2.2 mm; standard deviation ±0.6 mm).

 All premature infants that resulted with a PDA during 48-96 hours of life were treated for three consecutive days with three doses of ibuprofen (dosages and routes described above). 24 hours after the third dose, an echocardiography was performed, and if PDA persisted, a second course of ibuprofen treatment with three other doses was given. Patients with persistent PDA even after the second course were surgically treated. As a result, the closure time was 4 days for the first responsive group (53 patients [30 treated orally and 23 intravenously], see graphics below); 7 days for the responsive group undergoing the second course of treatment (15 patients in total); and the time of surgery for completely non-responsive patients (3 patients undergoing ligation). 

 After the first course of the treatment, PDA was closed in 30 (83.3%) of the patients assigned to the oral ibuprofen group versus 23 (71.8%) of those enrolled in the intravenous ibuprofen group. Six (16.6%) patients in the oral ibuprofen group required a second course of drug therapy, compared with 9 (28.1%) in the intravenous ibuprofen group. 15 patients needed a second treatment course and they had all (100%) clinical signs of infection and positive blood culture. The cumulative closure rates were higher in both groups, and only three (9.3%) patients in the intravenous ibuprofen group had surgical ligation. There was no reopening of the ductus after closure was achieved. Baseline characteristics were similar between the two groups in the first 96 hours (Table 1). In the evaluation of renal tolerance, none of the patients had oliguria.

 The serum creatinine levels and plasma blood urea nitrogen after the treatment did not differ significantly between the groups (Table 2).

## Discussion

Intravenous ibuprofen is not available in most countries (and in our country too), and is more expensive than the oral form. If oral ibuprofen was as efficient as intravenous ibuprofen with no greater adverse effects, the more simple administration and lower cost would be important advantages. Our study was designed with sufficient power for determining whether oral and intravenous ibuprofen treatments are equally efficacious and safe in PDA closure in premature infants with RDS. Our results showed oral ibuprofen to be effective and safe in PDA closure, with 30 of our 36 (83.3%) study infants achieving a successful outcome. The rate of closure in the group assigned to intravenous ibuprofen was similar to rates previously reported^[^^4^^,^^11^^]^. Some trials on the use of oral ibuprofen for closure of PDA have been recently published^[^^14^^,^^15^^,^^21^^]^.

**Table 1:** Baseline characteristics of the low birth weight preterm infants in oral vs intravenous ibuprofen therapy

**Variable**		**Oral (n = 36)**	**Intravenous (n= 32)**
**Gestational age, weeks**	28.1 – 30 weeks	19 (52.7%)	18 (56.2%)
	30.1 – 32 weeks	17 (47.2%)	14 (43.7%)
**Birth weight, grams**	<750g n (%)	2 (5.5%)	0 (0%)
	751-1000g n (%)	7 (19.4%)	6 (18.7%)
	1001-1500g n (%)	15 (41.6%)	19 (59.3%)
	1501-2000g n (%)	12 (33.3%)	7 (21.8%)
**Gender**	Male, n (%)	22 (61.1%)	15 (46.8%)
	Female, n (%)	14 (38.8%)	17 (53.1%)
**Delivery by cesarean section, n (%)**		20 (55.5%)	14 (43.7%)
**Antenatal glucocorticoid treatment, number and percentage (%)**		28 (77.7%)	18 (56.2%)
**Perinatal asphyxia, n (%)**		11 (30.5%)	9 (28.1%)

**Table 2:** Biochemical values in two group low birth weight preterm infants in oral vs intravenous ibuprofen therapy

** Parameters**		**Oral group** **(n=36)**	**Intravenous ** **group (n= 32)**	***P. *** **value**
**Plasma blood urea nitrogen (mg/dl) (mean ± SD)**	Day 1	30.7 (14.8 )	30.4 (13.7)	.90
	Day 2	30.3 (14.2)	30.6 (14.0)	.89
	Day 3	30.88 (7.76)	31.66 (9.90)	.68
**Mean plasma ** **creatinine (mg/dL) (mean±SD) **	Day 1	1.07 (0.24)	1.09 (0.24)	.06
	Day 2	1.20 (0.95)	0.97 (0.45)	.07
	Day 3	0.76 (0.48 )	0.79 (0.46)	.07
**Oligo/anuria (ml/kg/h)**	Day 1	0 (0%)	0 (0%)	
	Day 2	0 (0%)	0 (0%)	
	Day 3	0 (0%)	0 (0%)	
**Infection and need for a second treatment course**		6 (16.6%)	9 (28.1%)	
**Need for surgical ligation**		0 (0%)	3 (9.3%)	

All studies had small sample size. Aly^[^^16^^]^ in a randomized pilot study, reported that PDA was closed in 7 of 9 premature (≤35 weeks) infants given oral ibuprofen and in 10 of 12 premature infants given intravenous indomethacin.

 Fakhraee^[^^22^^]^ in a randomized study, reported that PDA was closed in all of 18 premature (≤34 weeks) infants given oral ibuprofen and in 15 of 18 premature infants given oral indomethacin (*P*>.05). Efficacy of oral ibuprofen compared with intravenous indomethacin, was reported by Supapannachart et al^[^^23^^]^ and Chotigeat et al^[^^24^^]^ as well. In nonrandomized open trials, Heyman et al^[^^25^^]^ and Cherif et al^[^^18^^]^ reported a ductal closure with oral ibuprofen respectively in 21 (95.4%) of 22 patients, 38 (95%) of 40 patients, and in 11 (84.6%) of 13 patients. The authors concluded that oral ibuprofen might constitute a feasible alternative in the treatment of PDA. Van Overmeire et al studied the efficacy of indomethacin and ibuprofen given to larger premature (≤32 weeks) infants at the age of 2-4 days. They reported that the closure rate was similar (66% and 70%, respectively) after the first course and that there was no significant difference in side effects, although ibuprofen was associated with significantly less impairment of renal function^[^^11^^,^^25^^]^. Two studies increase the number of infants randomized and expand the information about the safety and efficacy of oral ibuprofen in more mature VLBW (very low birth weight) infants^[^^19^^,^^20^^]^. We hope the same for our study. 

 Since renal tolerability of ibuprofen on renal function in the neonate is a major argument in favor of its use in the treatment of PDA^[^^19^^,^^20^^]^ our study expands our information about the safety and efficacy of oral ibuprofen in more mature VLBW infants. 

 Serum creatinine and urea levels in our patients were within normal range at all times, so there was no contraindication for a second dose of ibuprofen when it was needed. This might be an explanation for the higher rate of pharmacologic ductal closure observed in our study.

 Gonzales at al showed that late PDA episodes were more frequent in infants with infection than in those without infection and were associated with an increased risk of PDA closure failure. Furthermore, when both factors were temporally related, they further increased the risk of CLD^[^^5^^,^^7^^]^. 

 There are several limitations to our study. This was an open-label, one-arm study, and the physicians and nurses were aware of the nature of the study, although the cardiologist who supervised the echocardiographic studies was blinded as with regard to the status of the infants, and whether they were treated with oral ibuprofen or intravenous ibuprofen. On the other hand, being not a hemodynamic study, we could not obtain pressure values of the heart chambers (systolic and diastolic pressures, TR pressure gradients, pulmonary artery pressure); such a data collection would have surely enhanced the value of our conclusions. The exclusion of PDA patients whose ductal size was less than 1.5 mm might be another limitation, although several sources use such a cutoff^[^^4^^-^^6^^]^. This is not simply related to the fact that a smaller ductal size might be not important hemodynamically, and other authors do consider, although through the denomination ‘mild’, the hemodynamic importance even of a ductal size of less than 1.5 mm^[^^26^^]^. The problem of visualizing a patent ductus and thus differentiating it from a closed ductus when values are less than 1.5 mm remains however, a technical challenge^[^^27^^]^. This technicality might be overcome through using a 3D (three-dimensional) echocardiography, with higher spatial resolution and a better diagnostic accuracy^[^^28^^]^. 

 This is the first experience that we have had in Albania with ibuprofen (oral or intravenous) regarding the treatment of PDA in preterm infants.

## Conclusion

Our data indicate that, for preterm infants especially for LBW infants, the rate of early ductal closure was comparable and the adverse effects were fewer with oral ibuprofen in comparison to the intravenous route. Association of PDA with perinatal infection has a negative impact on the pharmacological closure of the ductus, increasing need for a second course and for surgical ligation. The oral form was as safe as the intravenous form in terms of renal tolerance. Larger comparative studies are needed to validate these findings.
